# Impact of Intraoperative Fluid Balance and Norepinephrine on Postoperative Acute Kidney Injury after Cystectomy and Urinary Diversion over Two Decades: A Retrospective Observational Cohort Study

**DOI:** 10.3390/jcm12134554

**Published:** 2023-07-07

**Authors:** Markus Huber, Marc A. Furrer, François Jardot, Dominique Engel, Christian M. Beilstein, Fiona C. Burkhard, Patrick Y. Wuethrich

**Affiliations:** 1 Department of Anaesthesiology and Pain Medicine, University Hospital Bern, 3010 Bern, Switzerland; markus.huber@insel.ch (M.H.); marcalain.furrer@outlook.com (M.A.F.); francois.jardot@insel.ch (F.J.); dominique.engel@insel.ch (D.E.); christian.beilstein@insel.ch (C.M.B.); 2 Department of Urology, University Hospital Bern, 3010 Bern, Switzerland; fiona.burkhard@insel.ch; 3 Department for Biomedical Research, University of Bern, 3010 Bern, Switzerland

**Keywords:** intraoperative fluid balance, norepinephrine, acute kidney injury (AKI), cystectomy

## Abstract

The use of norepinephrine and the restriction of intraoperative hydration have gained increasing acceptance over the last few decades. Recently, there have been concerns regarding the impact of this approach on renal function. The objective of this study was to examine the influence of norepinephrine, intraoperative fluid administration and their interaction on acute kidney injury (AKI) after cystectomy. In our cohort of 1488 consecutive patients scheduled for cystectomies and urinary diversions, the overall incidence of AKI was 21.6% (95%—CI: 19.6% to 23.8%) and increased by an average of 0.6% (95%—CI: 0.1% to 1.1%, *p* = 0.025) per year since 2000. The fluid and vasopressor regimes were characterized by an annual decrease in fluid balance (−0.24 mL·kg^−1^·h^−1^, 95%—CI: −0.26 to −0.22, *p* < 0.001) and an annual increase in the amount of norepinephrine of 0.002 µg·kg^−1^·min^−1^ (95%—CI: 0.0016 to 0.0024, *p* < 0.001). The interaction between the fluid balance and norepinephrine levels resulted in a U-shaped association with the risk of AKI; however, the magnitude and shape depended on the reference categories of confounders (age and BMI). We conclude that decreased intraoperative fluid balance combined with increased norepinephrine administration was associated with an increased risk of AKI. However, other potential drivers of the observed increase in AKI incidence need to be further investigated in the future.

## 1. Introduction

Radical cystectomies with urinary diversions are associated with a high postoperative complication rate (up to 60%). In addition, preoperative impaired renal function due to patient comorbidities, preoperative tumour-associated postrenal obstructions or nephrotoxic medication is not uncommon. Postoperative acute kidney injury (AKI) is associated with increased morbidity, mortality and length of hospital stay (LOS) following many types of major cardiac and non-cardiac surgeries [1,2,3,4,5,6,7]. Little has been published on postoperative AKI after cystectomies [8].

During the last decades, intraoperative fluid administration has evolved from a liberal to a more restrictive regimen and has become a cornerstone of ERAS^®^ protocols to avoid salt and water overload [9]. While perioperative hydration protocols aiming for a zero fluid balance in patients undergoing major abdominopelvic surgery have led to a significant overall reduction in both severe and gastrointestinal complications [10,11,12,13,14], a retrospective multicentre series indicated that the increasing incidence of AKI observed over the last years was correlated with restrictive fluid administration [15]. In addition, the focus on intraoperative normotensive patients to protect renal function has been established. Using predictive artificial devices has already been discussed with disputable outcomes [16,17,18].

Therefore, concerns have arisen that restrictive perioperative hydration and the supportive use of vasopressors potentially cause hypovolemia, which in turn can lead to renal dysfunction [19]. On one hand, perioperative urinary output is still widely considered a surrogate of renal function and additional fluid boluses are generally administered to reverse oliguria despite a lack of evidence thereof [14,20,21]. On the other hand, the administration of norepinephrine has been linked to impaired micro-circulation, potentially resulting in organ dysfunction and affecting renal function. If assessment of capillary refill time has been proven to be able to early detect impaired microcirculation intraoperatively, it has been implemented widely [22,23].

Postoperative AKI has been associated with increased morbidity, mortality and LOS in multiple major cardiac and non-cardiac procedures [1,2,3,4,5,6]. However, little is known about the impact of the intraoperative administration of colloids and crystalloids and the use of vasopressors such as norepinephrine on postoperative AKI after cystectomies.

The goal of this study was to determine the association between intraoperative hydration and vasopressor administration with postoperative AKI in patients undergoing cystectomies and urinary diversions and particularly to investigate whether there is an interaction between these two entities with respect to AKI. In addition, we describe the trends in fluid and norepinephrine administration over 20 years in a high-case-load, single tertiary referral centre for cystectomy and urinary diversion and identify risk factors for AKI using a multivariable logistic regression.

## 2. Materials and Methods

This observational study reports on a consecutive cohort from a single tertiary centre and is in accordance with the STROBE statement. Ethical approval for this study was provided by the Ethical Committee of Canton Bern, Switzerland (KEKBE 2016-00660, Chairperson Professor C. Seiler) on 2 June 2016, and the need for informed consent was waived.

### 2.1. Study Population

All patient data were collected from a prospectively maintained cystectomy database, which fully complies with the federal act on research involving humans. Patients and procedures from 1 January 2000 to 30 June 2020 were extracted from the database and completed from the patients’ paper charts and anaesthetic protocols.

### 2.2. Surgical Technique and Perioperative Management

At our institution, open cystectomies with urinary diversions and anaesthesia induction and maintenance have been performed following the same standardized surgical technique for the last 20 years as described previously [24,25].

After insertion of an epidural catheter at the low thoracic level, induction of anaesthesia (fentanyl 2 µg·kg^−1^ bolus, propofol 2 mg·kg^−1^ and rocuronium 0.6–0.9 mg·kg^−1^ intravenously to facilitate endotracheal intubation) and maintenance of anaesthesia with halogenics were performed according to our daily practice.

During the observation period, fluids administered were lactated Ringer’s solution and 6% hydroxyethyl starch 130/0.4 (starting from 2007) or 4% gelatin (before 2007). As of 2007, we aimed for a protocol-driven systematic restrictive fluid administration using a pre-emptive continuous administration of norepinephrine at around 1 to 2 µg·kg^−1^·h^−1^ combined with a fluid maintenance rate of approximately 1 to 3 mL·kg^−1^·h^−1^ of crystalloids beginning after initiation of the epidural segmental blockade and anaesthesia induction. The blood loss was primarily replaced with crystalloids. Albumin 20% up to 3 mL·kg^−1^ could be administered at the discretion of the anaesthesiologist in charge in case of severe blood loss (>20% of the estimated blood volume). Packed red blood cells (PRBCs) were transfused if haemoglobin values decreased to <80 g·L^−1^. Assessment of urinary output was not feasible during surgery because of external derivation of the ureter.

Postoperative hydration consisted of 1000 mL of Ringer’s solution and 500 mL of glucose 5% daily until resumption of normal food intake [26]. In case of hypotension, an additional bolus of 250–500 mL of Ringer’s solution was administered. Immediately after surgery, the patients were offered clear fluids. A peroral liquid diet and active mobilization were started on postoperative day (POD) 1.

### 2.3. Data Collection and Outcome Measures

The data collected included all relevant baseline characteristics, preoperative urine drainage due to postrenal obstruction and oral medication. Surgical factors recorded included the duration of surgery, type of urinary diversion, blood loss and the need for blood transfusion. Anaesthetic factors recorded included the type and total amount of fluid administered (crystalloids and colloids in mL), intraoperative fluid balance (difference between amount of fluid administered (IN: crystalloid, colloid and blood transfusion in mL) and fluid loss (OUT: blood loss in mL)) and differentiation in mL per kg bodyweight per hour (mL·kg^−1^·h^−1^). Likewise, continuous administration of norepinephrine (µg·kg^−1^·h^−1^) was recorded.

### 2.4. Primary Endpoint

The primary endpoint was the incidence of postoperative AKI. AKI was defined according to the Kidney Disease: Improving Global Outcomes (KDIGO) classification based on changes in plasma creatinine levels [27,28]. In addition, ureteral stents were always left in place for >6 days postoperatively, thus reducing the risk of bias due to postrenal obstruction. Urine output data were not used for AKI diagnoses because of inconsistent records.

### 2.5. Statistical Analysis

In terms of summary statistics, categorical variables were presented by means of counts and percentages and numerical variables by means of mean and standard deviation in cases of normally distributed variables and by median and interquartile range otherwise. Unadjusted, exploratory landmark analyses with respect to the primary outcome AKI for baseline, intraoperative and postoperative variables were computed using the chi-square test or Fisher’s exact test for qualitative variables with either Student’s *t*-test (normally distributed) or the Mann–Whitney test (skewed) for quantitative variables.

We used a data-driven approach to illustrate the various relationships between the variables in our dataset. First, all pairwise correlation coefficients (Pearson’s correlation coefficients for numerical variables and tetrachoric correlation coefficients for binary variables) were computed. Second, univariable associations as expressed by the logarithmic odds ratio of each variable with respect to the primary outcome of AKI were computed using logistic regression. To facilitate the comparison between different numeric variables of different scales and value ranges, the numerical variables were scaled with respect to their mean and standard deviation for univariable logistic regression. The correlation matrix was visualized with a network graph and a directed force layout [29], in which similar variables were grouped together and the arc width represented the strength of the pairwise correlations. The size and colour of the network nodes indicate the sign and magnitude of the logarithmic odds ratios. The network graphs are shown for two clinically chosen time periods (2000 to 2007 and 2008 to 2020), due to changes in fluid regimes avoiding synthetic colloids and the implementation of continuous administration of low-dose norepinephrine.

Networks provide a versatile tool for visualizing and investigating patterns in patient- and treatment-related variables. Given the subtle shifts in the network over time particularly with respect to fluid management, we continued to quantitatively examine the temporal trends in the variables in more detail and quantify their relationship with the incidence of AKI over the observational period.

Descriptive time series of different intraoperative fluids and norepinephrine administration were computed with a general additive model (GAM) to account for possible non-linear features over time. We adjusted for age, BMI and use of antihypertensives, beta-blockers, statins and platelet aggregation inhibitors as well as for hydronephrosis, nephrostomy, double-J stent (referred to as DJ stent in the figures and tables) and Charlson score.

The adjusted associations of both intraoperative fluids and norepinephrine with AKI risk were computed using logistic regression, in which a cubic spline approach for each type of fluid was chosen to allow for possible non-linear associations; a fluid–vasopressor interaction was omitted. We further computed a multivariable logistic regression model accounting for the interaction between total fluid balance and norepinephrine interaction.

The selection of variables used for confounder adjustment was based on clinical considerations and clinical practice; no variable selection procedure (i.e., stepwise variable selection) was performed as this might lead to biases in the estimated regression coefficients [30].

To translate the associations into clinical risk prediction, a nomogram was computed based on a multivariable logistic regression model (Supplementary Materials). Note, however, that an arbitrary—albeit clinically motivated—threshold value of 0.05 µg·kg^−1^ min^−1^ for the norepinephrine administration was chosen to translate the non-linear fluid–vasopressor interaction to the nomogram. Model performance was examined using the area under the receiver operating characteristic curve (AUROC) and Brier score; model calibration was assessed using a calibration curve.

In terms of missing data, a complete case analysis was performed.

Statistical significance was set at <0.05, and all computations were performed with R version 4.0.2 [31]. The multivariable logistic regression featuring cubic splines for the fluid balance as well as the nomogram were computed with the *rms* package [32]. The network graphs were computed with the *ggraph* package [33]. The computations of all pairwise correlation coefficients were performed with the *psych* package [34]. Estimated marginal means were computed with the *emmeans* package [35].

## 3. Results

The patients with insufficient follow-ups (missing data, N= 4) or who died ((N = 1) within 72 h after surgery) were excluded from the analysis, leaving 1483 (data missing: 0.34%) for the study.

Table 1 presents the demographic characteristics and comorbidities of the study cohort. In terms of the unadjusted group comparisons with respect to the outcome of acute kidney injury, the patients differed with respect to BMI, antihypertensives, platelet aggregation inhibitors, the incidence of coronary and valvular heart disease, ASA physical scores and age-adjusted Charlson comorbidity scores.

Anaesthesia-related details regarding the administration of intraoperative fluids and vasopressors are shown in Table 2. The median duration of surgery was 386 (IQR: 340 to 430) min with a median blood loss of 1000 (IQR 700 to 1400) mL. Over the course of the observational period, 28.2% of the patients received colloids, 25.8% received packed red blood cells (PRBCs) and 13.8% received fresh frozen plasma (FFP) with median amounts of 1.3 (IQR: 0.7 to 2.3) mL·kg^−1^·h^−1^ (colloids), 1.1 (IQR: 0.8 to 1.7) mL·kg^−1^·h^−1^ (PRBCs) and 1.2 (IQR: 0.8 to 1.6) mLkg^−1^h^−1^ (FFP), respectively. Norepinephrine was administered in 69.7% of the patients with a median dose of 0.04 (IQR: 0.02 to 0.06) µg·kg^−1^·min^−1^. Overall, median amounts of 4.5 (IQR: 3.2 to 6.0) mL·kg^−1^·h^−1^ of crystalloids and 5.1 (IQR: 3.6 to 7.6) mL·kg^−1^·h^−1^ of total fluids were administered with a fluid balance (in–out) of 2.9 (IQR: 1.6 to 4.9) mL·kg^−1^·h^−1^, in which the quantity of each differed in patients with and without AKI (all *p*-values <0.001).

**Figure 1:**
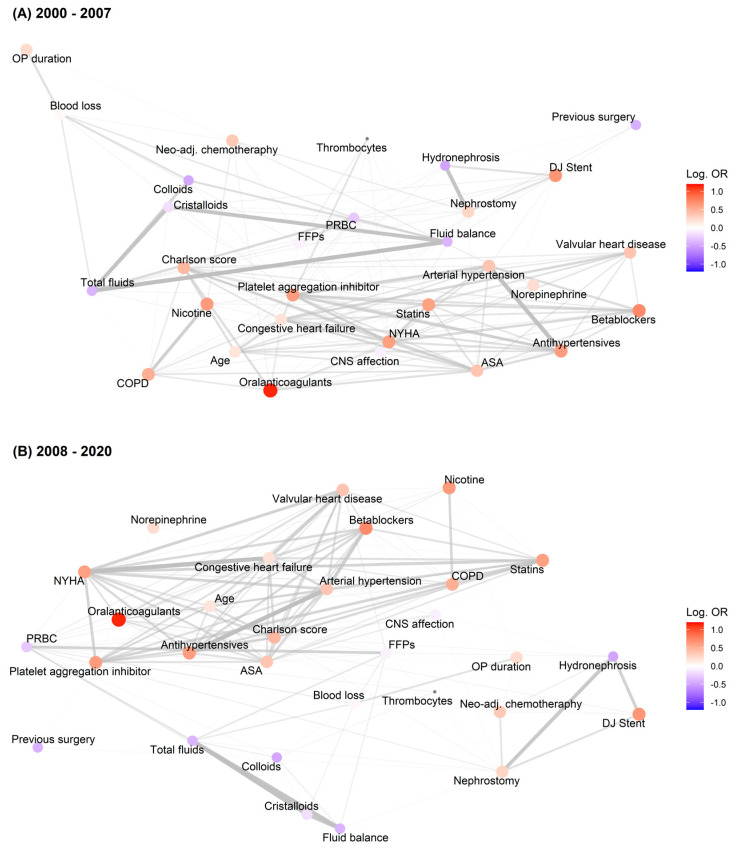
Multivariable cohort characteristics in two clinically related time periods visualized by means of a force-directed graph layout. Panel (**A**) refers to the time period 2000–2007, whereas panel (**B**) refers to the years 2008–2000. The widths of the arcs in the network represent the absolute correlation coefficient between two variables. To facilitate the visual inspection of the network, only arcs with absolute correlations above 0.1 are shown. The size and colour of the network nodes represent the univariable, unadjusted logarithmic odds ratios (ORs) of each scaled variable with respect to the primary outcome of this study (acute kidney injury, AKI). To facilitate the inter-variable comparison, the variables were scaled with respect to their means and standard deviations prior to the computation of the logarithmic ORs.

The variables presented in Table 1 and Table 2 illustrate both patient-specific and intervention-related variables with important clinical relationships. Figure 1 depicts these multivariable cohort characteristics in two clinically related time periods visualized using a force-directed graph layout. The force-directed layout allows for the clustering of similar variables and discovering subtle relationships among the variables in the dataset, in which the blue nodes represent protective factors (odds ratio < 1), whereas the red-coloured nodes represent risk factors (odds ratio > 1) with respect to AKI. The time periods are the years 2000–2007 and 2008–2020 and reflect a change in anaesthesia-related practices at our institution, notably with respect to the administration of synthetic colloids and the use of norepinephrine.

During the years 2000–2007, there were strong associations between colloids, crystalloids, PRBC, FFPs and the fluid balance as can be seen by means of the arc widths. In addition, comorbidities and risk factors were strongly associated. In the second period (2008–2020), there was a noticeable shift in terms of the following associations: the comorbidities were grouped closer together, and the intraoperative fluids, for example the PRBCs and FFPs, were further away from the total fluid balance and are less correlated (they have thinning arc widths).

Moreover, norepinephrine was further away from the total fluid balance than in the previous period.

**Figure 2:**
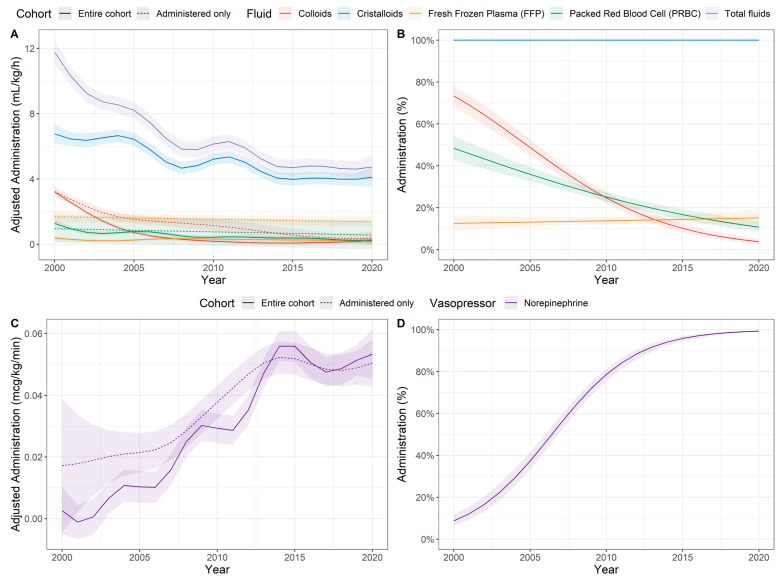
Time evolution of confounder-adjusted fluid management (**A**,**B**) and norepinephrine administration (**C**,**D**) based on a general additive regression model (GAM), adjusted for age, BMI, ASA score, antihypertensives, betablockers, statins, platelet aggregation inhibitors, hydronephrosis, nephrostomy, DJ stents and Charlson scores. The time evolution is shown both for the entire cohort and for the subgroup of patients who received fluids or vasopressors (referred to as “administered only”). The time evolutions of the percentage of patients who received the corresponding fluids or the vasopressors are illustrated in panels (**B**,**D**) and are based on a binomial regression model. Solid lines are the mean estimate, whereas the shaded band denotes the 95% confidence intervals. Estimated marginal means are shown for a 70-year-old patient with a BMI of 25 kg m^−2^, ASA status of 3 and Charlson score of 5.

The time evolutions of the confounder-adjusted fluid management (Figure 2A,B) and noradrenalin administration (Figure 2C,D) are shown in Figure 2 for both the entire cohort and the subgroup of patients that received fluids or vasopressors, respectively, thus accounting for the zero-inflated property of these clinical variables. Overall, a more restrictive intravenous fluid administration and the use of colloids accompanied by an increased administration of norepinephrine were observed during the last decade.

The largest decrease in absolute terms was observed for colloids and crystalloids (Figure 2A), whereas the largest decrease in relative terms was dominated by colloids and PRBCs (Figure 2B). A decrease in intravenous fluid administration (−0.29 mL·kg^−1^·h^−1^, 95%—CI: −0.31 to −0.26 mL·kg^−1^·h^−1^, *p* < 0.001) and the fluid balance (−0.24 mL·kg^−1^·h^−1^, 95%—CI: −0.26 to −0.22 mL·kg^−1^·h^−1^, *p* < 0.001) was observed over the observation time. The administration of colloids decreased from 98.0% in all patients during the year 2000 to 35.9% in 2020; the administration of PRBCs decreased from 69.4% to 12.8% during the study period.

In contrast, the median administered dose of norepinephrine increased from 0.02 µg·kg^−1^·min^−1^ in 2000 to 0.05 µg·kg^−1^min^−1^ in 2020, corresponding to an average linear increase of 0.002 µg·kg^−1^·min^−1^ (95%-CI: 0.0016 to 0.0024 µg·kg^−1^·min^−1^, *p* < 0.001) per year. In addition, the percentage of patients receiving norepinephrine increased annually by 5.1% (95%—CI: 4.1% to 6.2%, *p* < 0.001). Note that the unadjusted values are shown here for illustration purposes because the adjusted values in Figure 2 depend on the choice of the reference category for all the covariates, for example, the comorbidities.

**Figure 3:**
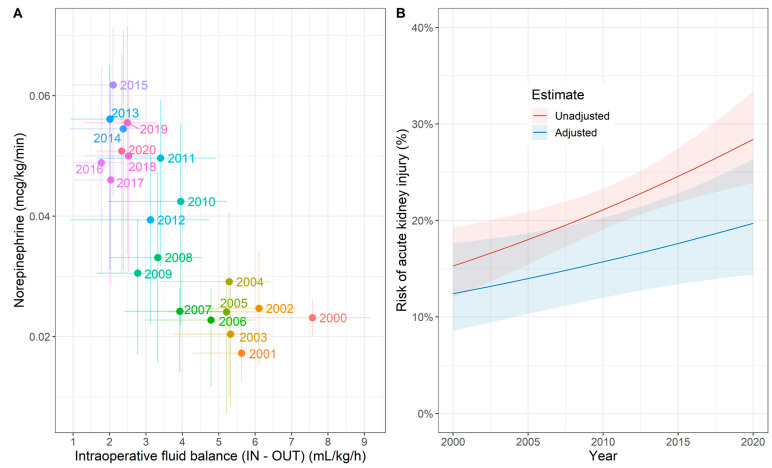
(**A**) The time evolution of fluid and vasopressor treatment over the past 20 years. Coloured dots represent the annual mean norepinephrine and fluid balance, respectively, and the lines represent the associated 95% confidence intervals. Only patients who received norepinephrine are shown. (**B**) Unadjusted and adjusted risk of acute kidney injury (AKI) over the past 20 years, where the line shows the mean estimate and the shaded bands denote the 95% confidence intervals. This panel shows the adjusted AKI risk for a 70-year-old patient with a BMI of 25 kg·m^−2^, ASA status 3 and Charlson score of 5.

The joint time evolutions of the fluid and vasopressor treatments over the past 20 years are illustrated in Figure 3A, highlighting a significant shift from a high fluid balance—low norepinephrine regime in the early years towards a low fluid balance—high norepinephrine regime in recent years. Figure 3B depicts the incidence of AKI during the study period. Overall, the incidence of postoperative AKI was 21.6% (95%—CI: 19.6% to 23.8%), and 91.0% (95%—CI: 87.3% to 93.9%) of the patients diagnosed with AKI were in AKIN stage 1. The incidence of AKI was 12.2% (95%—CI: 4.6% to 24.8%) in 2000 and increased on average by 0.6% (95%—CI: 0.1% to 1.1%, *p* = 0.025) over the following two decades. Importantly, the adjusted increase in the AKI incidence was lower when adjusted for confounders (Figure 3B). No patients required haemodialysis in the early postoperative period.

**Figure 4:**
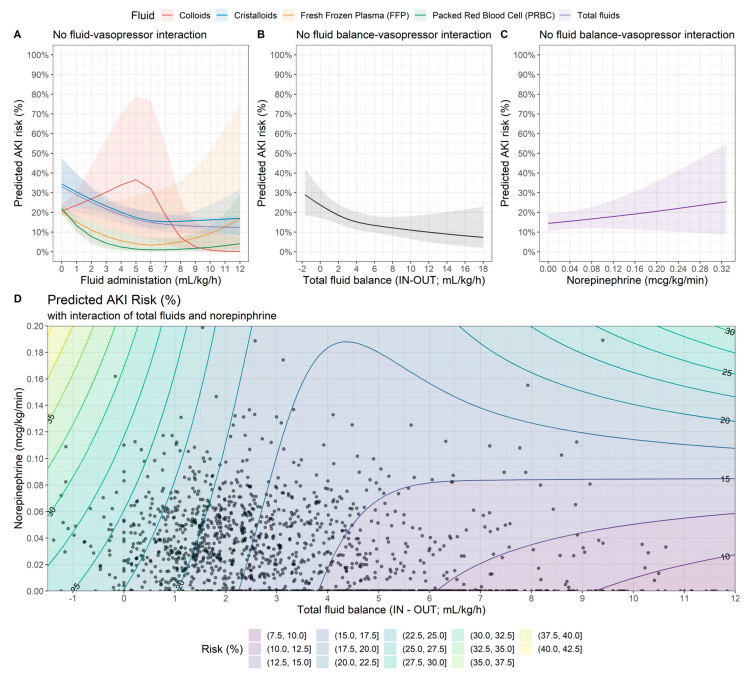
Adjusted risk of AKI as a function of fluid management (**A**), vasopressor (**B**) and fluid balance (**C**) without considering fluid–vasopressor interaction. In panels (**A**,**B**), norepinephrine was set at 0.05 µg·kg^−1^·min^−1^. (**D**) Adjusted risk of AKI accounting for fluid–vasopressor interaction. The risk predictions are based on a multivariable logistic regression model in which the fluid variable was modelled by means of a restricted cubic spline with 3 knots. Solid dots represent the treatment constellation for individual patients. Total fluid balance is shown up to 12 mL·kg^−1^·h^−1^ and norepinephrine is shown up to ≤ 0.2 µg·kg^−1^·min^−1^. Only 25 patients (1.7%) had values outside these ranges. Estimated marginal means are shown for a 70-year-old patient with a BMI of 25 kg·m^−2^, an ASA status of 3 and Charlson score of 5.

The confounder-adjusted predicted AKI risk as a function of fluid and vasopressor administration is shown in Figure 4. In terms of the total fluid balance, the AKI risk was strongly reduced to approximately 2 mL·kg^−1^·h^−1^, after which point the risk reduction was less pronounced (Figure 4B). We found that the AKI risk increased approximately linearly with norepinephrine administration; however, the estimates were more uncertain with increasing norepinephrine doses (Figure 4C). Figure 4D illustrates the AKI predicted risk accounting for the interaction between the total fluid balance and norepinephrine administration for a particular choice of reference values for confounders. The figure shows the observed and predicted values in the clinically relevant range of up to total fluid balances of 12 mL·kg^−1^·h^−1^ and norepinephrine administrations up to 0.2 µg·kg^−1^·min^−1^; only 25 patients (1.7%) featured values beyond these limits. We constrained the ranges of the figures in order to limit the amount of extrapolation in the total fluid balance vs. vasopressor parameter space.

We found that the AKI risk features a distinct U-shaped valley: for a total fluid balance in the range of about 3–5 mL·kg^−1^·h^−1^, the AKI risk only slightly increases with increasing norepinephrine doses, thus representing the trough in the “AKI risk valley”. For the lower (and negative) total fluid balance, the AKI risk strongly increases as a function of norepinephrine administration. For the total fluid balances higher than ~5–7 mL·kg^−1^·h^−1^, the AKI risk also increases with higher norepinephrine administration. The dependency of this risk increase on the total fluid balance was less pronounced than that for very low total fluid balances. Overall, Figure 4 demonstrates a strong interaction between total fluid and vasopressor administration with a strong impact on the AKI risk.

To translate these results into clinical practice, we computed a nomogram based on a multivariable logistic regression model; Supplementary Figures S1 and S2 illustrate the nomogram and its calibration by means of a calibration curve. Importantly, the nomogram demonstrates the “U-shaped” risk profile for the interaction of the total fluid balance with a high norepinephrine administration (>0.05 µg·kg^−1^·min^−1^). The model demonstrates only a medium performance with an AUROC of 0.67 and a Brier score of 0.16. The independent risk factors were BMI (*p* < 0.001), the use of antihypertensives (*p* < 0.001) and the age-adjusted Charlson score (*p* = 0.012).

## 4. Discussion

In this monocentric retrospective cohort including a standardized high-risk surgical intervention over an observation period of two decades, AKI occurred in 21.6% of the patients undergoing cystectomies and urinary diversions. Almost 90% of the patients with postoperative AKI had AKIN grade 1. We found that a moderate fluid balance combined with a low rate of norepinephrine administration in the early 2000s was associated with a low risk of AKI.

During the observation period, we observed a shift to lower fluid balance combined with a moderate to high dosage of norepinephrine, resulting in an increased risk of postoperative AKI. This observation was confirmed after adjusting for confounding factors. Overall, the incidence of AKI was 12.2% in 2000 and increased by an average of 0.6% per year over the following two decades. In the patients with early postoperative AKI, their renal function remained impaired for more than five years compared to that in the patients without AKI. However, if assessed at 5 years postoperatively, it remains questionable whether the median GFR values between 63 mL·min^−1^ (AKI patients) and 74 mL·min^−1^ are of clinical relevance, as both values qualify as CKD grade 2.

Over the course of the two decades, we observed a more restrictive administration of intravenous fluid and an increase in the number of patients treated with norepinephrine, as well as an increase in norepinephrine administered intraoperatively. Of relevance, we found an association between decreased the intraoperative administration of intravenous fluids, number of patients treated with norepinephrine and amount of norepinephrine administered with AKI events. This is in line with numerous results from retrospective series and the conclusion of the RELIEF trial [36]. Here, we contribute new evidence that a restrictive intraoperative fluid balance, as a result of the difference between fluid administration and blood loss, was associated with increased AKI events. A nomogram containing all relevant variables was designed.

Besides unadjusted fluid administration, fluid balance and norepinephrine, additional parameters associated with AKI were BMI, the severity of comorbidities as illustrated by the by age-adjusted Charlson comorbidity score, ischemic and valvular heart disease, hypertension, the duration of surgery and preoperative renal function.

In this cohort, the risk of AKI showed a U-shaped association with respect to individual fluid administration except for colloids: overall, the risk of developing acute kidney injury increased if the total intravenous fluid administration was less than or above the range of approximately 5–8 mL·kg^−1^·h^−1^ (Figure 4A). However, if the total intraoperative fluid balance was taken into account, we found an association between low fluid balance and the incidence of AKI, the U-shaped association disappeared and the increased risk of AKI was nearly linear if the fluid balance was reduced from 12 to 5 mL·kg^−1^·h^−1^ (Figure 4B). If the adjusted risk of AKI for intravenous fluid administration and norepinephrine interaction was considered, the risk of AKI was strongly enhanced by concomitant high-dose norepinephrine administration. For example, if the total fluid balance was around 0 mL·kg^−1^·h^−1^, the risk of AKI was estimated at 25% if no norepinephrine was administered (subject to the particular choice of reference values for the covariates) as shown in Figure 4D. However, this risk increased to 30% when norepinephrine was administered at a rate of 0.1 µg·kg^−1^·min^−1^. Here, once again, the U-shaped form could be detected which implies that the risk increase is either due to a change in the total fluid balance or norepinephrine administration depending on the specific values of the quantity that is held constant.

Considering the effect sizes in the multivariable logistic regression model and their representations in the nomogram (Supplementary Materials), there might be other first-order predictors, such as BMI and the severity of comorbidities (i.e., Charlson score). Their interactions with the amount of fluid administered and vasopressor management should be investigated in more detail to explain the observed increase in the incidence of AKI. In this context, further statistical concepts, such as causal inferences, need to be adopted to move from a purely associational analysis framework of the effect of fluid–vasopressor interaction on postoperative renal failure towards a more detailed understanding of cause and effect in this setting [37].

### Strength and Limitation

This study has several limitations, including potential bias from unmeasured risk factors and some missing values (<1%). Additionally, despite the use of a multiple regression analysis and risk adjustment, the potential for residual confounding factors could not be eliminated entirely.

We explicitly emphasise here that large parts—in particular, extreme values—of the derived U-shaped risk curve that accounts for the total fluid balance–norepinephrine interaction are based on extrapolated values from a fitted multivariable logistic regression model and that these extreme regions might not be encountered in routine clinical practice.

Another issue is that it is still unclear if and to what extent creatinine is reabsorbed by the ileum used for urinary diversions, especially in the early postoperative period, and whether this reabsorption is clinically relevant. It was also impossible to diagnose AKI based on urinary output for the following reasons: intraoperative diuresis cannot be assessed due to the externalization of the ureters during surgery and postoperative urine output can be biased due to polyuria (e.g., after resolving pre-existing postrenal obstructions). However, urine output is not mandatory for the assessment of AKI. Urine output is still used as a surrogate for renal function to guide perioperative fluid therapy. Additional fluid administration to reverse oliguria and maintain urine output above a specific predefined threshold is proposed in daily practice. However, oliguria does not always occur because of decreased renal perfusion pressure or glomerular perfusion and therefore cannot be prevented by fluid administration. Surgical-stress-induced perioperative neuro-hormonal regulation is probably fluid unresponsive, and administering fluids to increase urine output may lead to fluid overload without any renal benefits [14,18]. Finally, our data are generated from a high-case-load centre specialized in this kind of surgery; whether these results can be generalized remains speculative.

One additional limitation is the absence MAP data, as it has been shown that hypotension (having an MAP below 65 mmHg) could be associated with an increase in the AKI rate. However, Chiu et al. recently showed that a “cosmetic” correction of hypotension, for example using vasopressors, is not per se protective against AKI. It must be noted that an MAP is not the ultimate target for protecting organ function. Perfusion pressure might be a more accurate parameter; however, it is much more difficult to monitor in a real-life clinical setting. Blood flow, cardiac output and vascular resistance must also be considered for optimal protective organ perfusion.

## 5. Conclusions

We observed a decreased administration of intravenous fluid, resulting in a decreased intraoperative fluid balance and the increased use of norepinephrine during cystectomies and urinary diversions over the last two decades. The decreased intraoperative fluid balance resulted in an increased incidence of AKI. However, as most AKI events are staged as AKIN grade 1, the clinical relevance of the observed AKI remains questionable.

## Figures and Tables

**Table 1:** Patients’ demographics and comorbidities. For group comparisons with respect to the binary outcome acute kidney injury (AKI), unadjusted *p*-values are shown.

	All Patients	Without AKI	With AKI	*p*
	N = 1483 (100%)	N = 1162 (78.4%)	N = 321 (21.6%)	
*Demographics*				
**Age** (years)	68.5 [60.9; 75.6]	68.4 [60.2; 75.6]	69.1 [63.5; 76.0]	0.07
**BMI** (kg·m^−2^)	25.8 [23.0; 28.7]	25.2 [22.6; 28.4]	27.1 [24.3; 30.4]	<0.001
*Comorbidities*				
**ASA physical status**				<0.001
1	31 (2.1%)	29 (2.5%)	2 (0.6%)	
2	733 (49.4%)	602 (51.8%)	131 (40.8%)	
3	682 (46.0%)	506 (43.5%)	176 (54.8%)	
4	37 (2.5%)	25 (2.2%)	12 (3.74%)	
**Antihypertensives** (Yes)	659 (44.4%)	467 (40.2%)	192 (59.8%)	<0.001
**Beta-blockers** (Yes)	307 (20.7%)	218 (18.8%)	89 (27.7%)	0.001
**Statins** (Yes)	346 (23.3%)	246 (21.2%)	100 (31.2%)	<0.001
**Oral anticoagulants** (Yes)	60 (4.1%)	43 (3.7%)	17 (5.3%)	0.26
**Platelet aggregation inhibitor** (Yes)	208 (14.0%)	145 (12.5%)	63 (19.6%)	0.002
**Nicotine** (Yes)	812 (54.8%)	630 (54.2%)	182 (56.7%)	0.47
**Arterial hypertension** (Yes)	756 (51.0%)	550 (47.3%)	206 (64.2%)	<0.001
**Coronary heart disease** (Yes)	362 (24.4%)	265 (22.8%)	97 (30.2%)	0.008
**Congestive heart failure** (Yes)	312 (21.0%)	237 (20.4%)	75 (23.4%)	0.28
**Valvular heart disease** (Yes)	107 (7.2%)	74 (6.4%)	33 (10.3%)	0.023
**COPD** (Yes)	298 (20.1%)	231 (19.9%)	67 (20.9%)	0.75
**CNS affection** (Yes)	207 (14.0%)	155 (13.3%)	52 (16.2%)	0.22
**Hydronephrosis** (Yes)	318 (21.4%)	248 (21.3%)	70 (21.8%)	0.92
**Nephrostomy** (Yes)	135 (9.10%)	103 (8.86%)	32 (9.97%)	0.62
**DJ stent** (Yes)	108 (7.28%)	82 (7.06%)	26 (8.10%)	0.61
**Charlson score** (age adjusted)	4.0 [0.0; 5.4]	4.0 [0.0; 5.0]	4.00 [2.0; 6.0]	<0.001
**Previous surgery** (Yes)	646 (43.6%)	513 (44.1%)	133 (41.4%)	0.42
**Neo-adjuvant chemotherapy** (Yes)	230 (15.5%)	172 (14.8%)	58 (18.1%)	0.18
*Type of surgery*				
**Year of surgery**	2011 [2006; 2015]	2010 [2006; 2015]	2012 [2007; 2016]	0.001
**Orthotopic ileal bladder substitute** (Yes)	655 (44.2%)	516 (44.4%)	139 (43.3%)	0.77
**Continent ileal reservoir** (Yes)	103 (7.0%)	85 (7.3%)	18 (5.6%)	0.35
**Ileal conduit** (Yes)	695 (47.0%)	537 (46.2%)	158 (49.2%)	0.37
**Ureterosigmoidostomy** (Yes):	3 (0.2%)	3 (0.3%)	0 (0%)	>0.99
**Ureterocutaneostomy** (Yes):	44 (3.0%)	35 (3.0%)	9 (2.8%)	0.99

**Table 2:** OP characteristics, fluid management, vasopressors and AKI. Unadjusted group comparisons with respect to the binary outcome acute kidney injury (AKI) are shown for exploratory purposes.

	All Patients	Without AKI	With AKI	*p*
	N = 1483	N = 1162	N = 321	
**OP duration** (min)	386 [340;430]	385 [338;425]	393 [345;442]	0.004
**OP blood loss** (mL)	1000 [700;1400]	1000 [700;1400]	1000 [700;1410]	0.379
*Intraoperative fluids*				
**Crystalloids** (mL·kg^−1^·h^−1^)	4.45 [3.23;5.96]	4.60 [3.35;6.16]	3.99 [2.84;5.35]	<0.001
**Colloids** (mL·kg^−1^·h^−1^)	0.00 [0.00;0.33]	0.00 [0.00;0.44]	0.00 [0.00;0.00]	0.017
**Colloids administered** (Yes)	418 (28.2%)	340 (29.3%)	78 (24.3%)	0.093
**Colloids administered** (mL·kg^−1^·h^−1^)	1.26 [0.70;2.32]	1.39 [0.75;2.45]	0.87 [0.50;1.61]	<0.001
**Packed red blood cells** (mL·kg^−1^·h^−1^)	0.00 [0.00;0.41]	0.00 [0.00;0.57]	0.00 [0.00;0.00]	0.009
**Packed red blood cells administered** (Yes)	382 (25.8%)	315 (27.1%)	67 (20.9%)	0.029
**Packed red blood cells administered** (mL·kg^−1^·h^−1^)	1.13 [0.76;1.67]	1.16 [0.78;1.68]	0.96 [0.61;1.55]	0.017
**Fresh frozen plasma** (mL·kg^−1^·h^−1^)	0.00 [0.00;0.00]	0.00 [0.00;0.00]	0.00 [0.00;0.00]	0.613
**Fresh frozen plasma administered** (Yes)	204 (13.8%)	162 (13.9%)	42 (13.1%)	0.762
**Fresh frozen plasma administered** (mL·kg^−1^·h^−1^)	1.20 [0.82;1.59]	1.22 [0.83;1.64]	1.04 [0.81;1.44]	0.181
**Thrombocytes** (mL·kg^−1^·h^−1^)	0.00 [0.00;0.00]	0.00 [0.00;0.00]	0.00 [0.00;0.00]	0.660
**Thrombocytes administered** (Yes)	7 (0.47%)	5 (0.43%)	2 (0.62%)	0.649
**Thrombocytes administered** (mL·kg^−1^·h^−1^)	550 [525;1800]	1100 [550;2500]	375 [312;438]	0.051
**Total fluids** (mL·kg^−1^·h^−1^)	5.09 [3.55;7.56]	5.27 [3.72;7.85]	4.25 [2.94;6.32]	<0.001
**Fluid balance (in–out)** (mL·kg^−1^·h^−1^)	2.89 [1.60;4.93]	3.06 [1.74;5.20]	2.35 [1.20;4.06]	<0.001
*Vasopressors*				
**Norepinephrine** (µg·kg^−1^·min^−1^)	0.03 [0.00;0.05]	0.02 [0.00;0.05]	0.03 [0.00;0.05]	0.004
**Norepinephrine administered** (Yes)	1034 (69.7%)	786 (67.6%)	248 (77.3%)	0.001
**Norepinephrine administered** (µg·kg^−1^·min^−1^)	0.04 [0.02;0.06]	0.04 [0.02;0.06]	0.04 [0.02;0.06]	0.755

## Data Availability

Data can be requested from the corresponding author with corresponding approval by the local ethics committee of the requesting institution.

## Supplementary Materials

The following supporting information can be downloaded at: https://www.mdpi.com/article/10.3390/jcm12134554/s1, Figure S1. Nomogram for predicting risk of acute kidney injury. Note that norepinephrine was categorized in 3 levels in order to account for the zero-inflated nature of norepinephrine administration. Figure S2. Calibration plot of the multivariable logistic regression model with AKI as binary outcome. Table S1. Laboratory measurements of creatinine and glomerular filtration rate. Table S2. Coefficient of the multivariable logistic regression model on which the nomogram is based on. Table S3. Coefficient of the multivariable logistic regression model without vasopressor and fluid-balance interaction and without a spline representation of the total fluid balance. Binary outcome is AKI. Table S4. STROBE Statement—Checklist of items that should be included in reports of cohort studies.
