# Supplementary material for: Impact of Intraoperative Fluid Balance and Norepinephrine on Postoperative Acute Kidney Injury after Cystectomy and Urinary Diversion over Two Decades: A Retrospective Observational Cohort Study
Source: J Clin Med. 2023 Jul 7;12(13):4554. doi: 10.3390/jcm12134554 (PMC10342260; doi:10.3390/jcm12134554)
Supplement: Supplementary file 1 [file jcm-12-04554-s001.zip › jcm-2480568-supplementary.pdf]

Supplementary Material: Impact of Intraoperative Fluid Balance and Norepinephrine on Postoperative Acute Kidney Injury after Cystectomy and Urinary Diversion over Two Decades: A Retrospective Observational Cohort Study

Markus Huber, Marc A. Furrer, François Jardot, Dominique Engel, Christian M. Beilstein, Fiona C. Burkhard, Patrick Y. Wuethrich

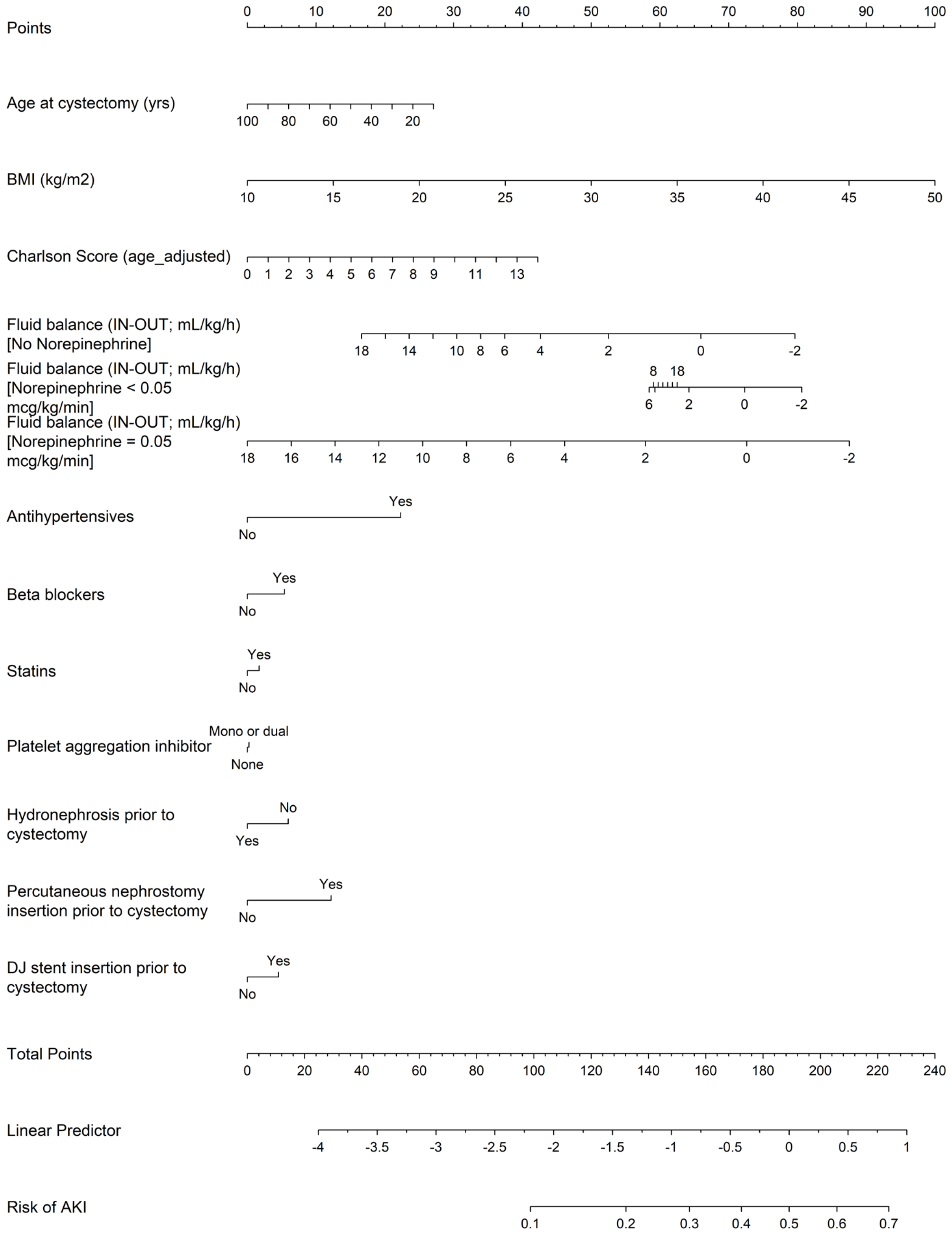

Figure S1. Nomogram for predicting risk of acute kidney injury. Note that norepinephrine was categorized in 3 levels in order to account for the zero-inflated nature of norepinephrine administration.

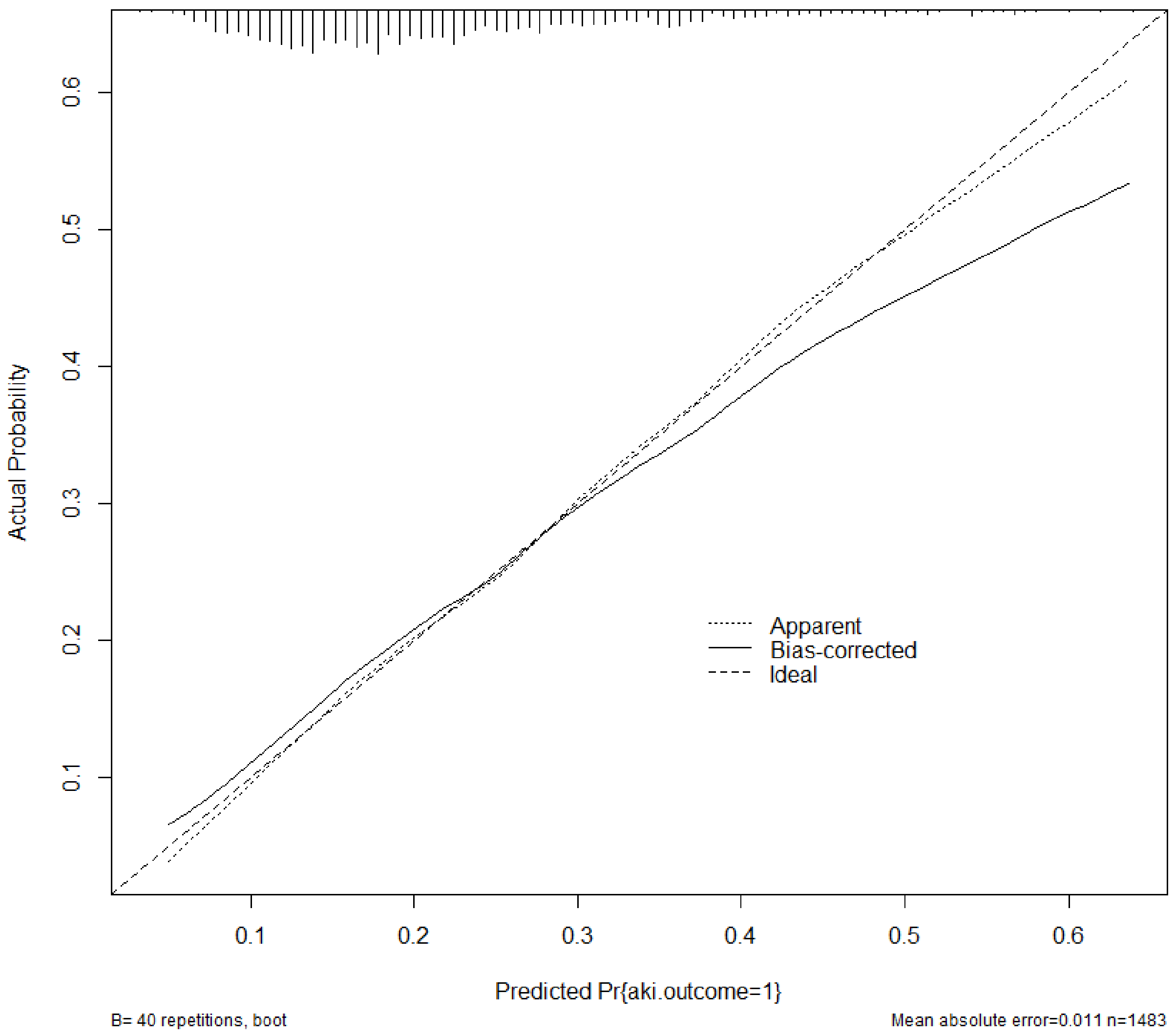

Figure S1. Calibration plot of the multivariable logistic regression model with AKI as binary outcome.

Table S1. Laboratory measurements of creatinine and glomerular filtration rate.

	All Patients	Without AKI	With AKI	*p*
	**N = 1483**	**N = 1162**	**N = 321**	
Laboratory measurements				
Creatinine (μmol/L)				
Preoperative [*n* = 1482]	86.0 [71.0;108.0]	85.0 [70.0;106.0]	92.0 [75.0;111.0]	0.01
1 month [*n* = 11,018]	86.0 [70.0;110.0]	83.2 [69.0;104.0]	96.5 [78.0;122.0]	<0.001
3 month [*n* = 1273]	85.0 [71.0;105.0]	83.0 [69.0;101.0]	95.0 [76.0;117.0]	<0.001
6 month [*n* = 1127]	89.0 [74.0;108.0]	87.0 [72.0;104.0]	96.0 [81.5;120.0]	<0.001
12 month [*n* = 972]	89.0 [74.0;109.0]	87.0 [73.0;104.0]	99.0 [80.2;128.0]	<0.001
12 month [*n* = 800]	91.0 [75.0;110.0]	89.0 [74.0;106.0]	98.0 [82.0;124.0]	<0.001
24 month [*n* = 715]	89.0 [76.0;112.0]	88.0 [74.0;107.0]	100 [81.0;123.0]	<0.001
36 month [*n* = 613]	88.0 [75.0;110.0]	87.0 [74.0;106.0]	100 [82.8;121.0]	<0.001

	All Patients	Without AKI	With AKI	*p*
	N = 1483	N = 1162	N = 321	
48 month [*n* = 524]	89.0 [76.0;108.0]	87.0 [75.0;105.0]	102 [83.0;130.0]	<0.001
60 month [*n* = 427]	89.0 [78.0;108.0]	88.0 [76.0;105.0]	100 [83.5;121.0]	0.002
72 month [*n* = 351]	90.0 [76.5;112.0]	88.0 [76.0;109.0]	104 [88.2;122.0]	0.002
GFR (mL/min)				
Preoperative [*n* = 1,482]	73.1 [55.3;93.1]	73.9 [55.9;94.0]	70.2 [52.5;90.0]	0.13
1 month [*n* = 1,1018]	85.6 [62.8;117]	88.0 [65.9;119]	77.5 [54.9;106]	<0.001
3 months [*n* = 1,273]	83.9 [63.3;108]	85.2 [65.1;111]	78.9 [56.6;103]	0.002
6 months [*n* = 1,127]	76.5 [57.7;98.5]	77.5 [59.6;99.4]	71.5 [52.9;95.0]	0.004
12 months [*n* = 972]	71.6 [54.5;89.1]	73.0 [56.4;91.0]	62.5 [46.4;82.9]	<0.001
12 months [*n* = 800]	71.9 [54.1;89.5]	73.0 [57.6;91.2]	63.0 [48.5;83.1]	0.001
24 months [*n* = 715]	71.8 [54.7;89.0]	73.2 [55.3;89.9]	64.2 [49.1;84.9]	0.015
36 months [*n* = 613]	74.0 [55.1;88.0]	75.4 [58.7;88.4]	64.6 [50.7;84.6]	0.020
48 months [*n* = 524]	72.6 [55.8;89.0]	74.3 [57.4;89.2]	63.8 [46.4;87.8]	0.012
60 months [*n* = 427]	72.5 [55.2;88.5]	73.9 [57.1;88.4]	62.8 [49.4;89.9]	0.12
72 months [*n* = 351]	73.0 [55.0;87.6]	73.7 [55.8;88.0]	66.8 [49.6;86.4]	0.11

Table S2. Coefficient of the multivariable logistic regression model on which the nomogram in the manuscript is based on (Figure 5).

Predictor	Log-odds	Standard Error	Wald Z	*p*
Intercept	−2.7294	0.7476	−3.65	0.0003
Age (years)	−0.0073	0.0069	−1.07	0.2866
BMI (kg/m²)	0.0609	0.0140	4.35	<0.0001
Antihypertensives (Yes)	0.5432	0.1490	3.65	0.0003
Beta-blockers (Yes)	0.1316	0.1641	0.80	0.4226
Statins (Yes)	0.0417	0.1662	0.25	0.8019
Platelet aggregation inhibitors (Yes)	0.0056	0.1938	0.03	0.9769
Hydronephrosis (Yes)	−0.1443	0.1901	−0.76	0.4481
Nephrostomy (Yes)	0.2966	0.2570	1.15	0.2484
DJ Stent (Yes)	0.1102	0.2599	0.42	0.6716
Charlson Score (age adjusted)	0.0734	0.0293	2.51	0.0122
Norepinephrine (≥ 0.05 μg kg^{-1} min^{-1} vs none)	0.1547	0.5702	0.27	0.7861
Norepinephrine (< 0.05 μg kg^{-1} min^{-1} vs none)	0.1622	0.5364	0.30	0.7624
Fluid balance (first difference in cubic splines)	−0.1664	0.1817	−0.92	0.3598
Fluid balance (second difference in cubic splines)	0.1228	0.2288	0.54	0.5914
Norepinephrine (>= 0.05 μg kg^{-1} min^{-1} vs none) × Fluid balance (first difference in cubic splines)	0.0654	0.2239	0.29	0.7701
Norepinephrine (< 0.05 μg kg^{-1} min^{-1} vs none) × Fluid balance (first difference in cubic splines)	−0.0152	0.2082	−0.07	0.9417

Norepinephrine (>= 0.05 μg kg⁻¹ min⁻¹ vs none) × Fluid balance (second difference in cubic splines)	−0.0148	0.3125	−0.05	0.9623
Norepinephrine (< 0.05 μg kg⁻¹ min⁻¹ vs none) × Fluid balance (second difference in cubic splines)	−0.0200	0.2874	−0.07	0.9445

Table S3. Coefficient of the multivariable logistic regression model without vasopressor and fluid-balance interaction and without a spline representation of the total fluid balance. Binary outcome is AKI.

Characteristic	OR [1]	95% CI [1]	*p*
Age at cystectomy (years)	0.99	0.98, 1.01	0.2
BMI (kg/m²)	1.06	1.03, 1.09	<0.001
Antihypertensives (Yes)	1.73	1.29, 2.31	<0.001
No	—	—	
Beta-blockers (Yes)	1.14	0.82, 1.56	0.4
Statins (Yes)	1.04	0.75, 1.44	0.8
Platelet aggregation inhibitors			
None	—	—	
Mono or dual	1.01	0.69, 1.47	>0.9
Hydronephrosis prior to cystectomy (Yes)	0.87	0.59, 1.25	0.5
Percutaneous nephrostomy insertion_prior to cystectomy (Yes)	1.33	0.80, 2.19	0.3
DJ stent insertion prior to cystectomy (Yes)	1.12	0.66, 1.84	0.7
Charlson Score (age_adjusted)	1.08	1.02, 1.14	0.011
Norepinephrine			
0 μg kg⁻¹ min⁻¹	—	—	
larger equal 0.05 μg kg⁻¹ min⁻¹	1.38	0.93, 2.05	0.11
smaller 0.05 μg kg⁻¹ min⁻¹	1.14	0.80, 1.64	0.5
Intraoperative fluid balance (IN – OUT; mL/kg/h)	0.92	0.86, 0.97	0.005

[1] OR = Odds Ratio, CI = Confidence Interval

Table S4. STROBE Statement—Checklist of items that should be included in reports of cohort studies.

	Item No	Recommendation	Page No
Title and abstract	1	(a) Indicate the study's design with a commonly used term in the title or the abstract	1
		(b) Provide in the abstract an informative and balanced summary of what was done and what was found	1
Introduction			
Background/ rationale	2	Explain the scientific background and rationale for the investigation being reported	2
Objectives	3	State specific objectives, including any prespecified hypotheses	2
Methods			
Study design	4	Present key elements of study design early in the paper	2
Setting	5	Describe the setting, locations, and relevant dates, including periods of recruitment, exposure, follow-up, and data collection	2
Participants	6	(a) Give the eligibility criteria, and the sources and methods of selection of participants. Describe methods of follow-up	2

		(b) For matched studies, give matching criteria and number of exposed and unexposed	na
Variables	7	Clearly define all outcomes, exposures, predictors, potential confounders, and effect modifiers. Give diagnostic criteria, if applicable	3
Data sources/ measurement	8 *	For each variable of interest, give sources of data and details of methods of assessment (measurement). Describe comparability of assessment methods if there is more than one group	3
Bias	9	Describe any efforts to address potential sources of bias	3–4
Study size	10	Explain how the study size was arrived at	4
Quantitative variables	11	Explain how quantitative variables were handled in the analyses. If applicable, describe which groupings were chosen and why	4
Statistical methods	12	(a) Describe all statistical methods, including those used to control for confounding	3–4
		(b) Describe any methods used to examine subgroups and interactions	4
		(c) Explain how missing data were addressed	4
		(d) If applicable, explain how loss to follow-up was addressed	na
		(e) Describe any sensitivity analyses	4
		Results	
Participants	13 *	(a) Report numbers of individuals at each stage of study—e.g. numbers potentially eligible, examined for eligibility, confirmed eligible, included in the study, completing follow-up, and analysed	4
		(b) Give reasons for non-participation at each stage	4
		(c) Consider use of a flow diagram	na
Descriptive data	14 *	(a) Give characteristics of study participants (e.g. demographic, clinical, social) and information on exposures and potential confounders	5 Table 1
		(b) Indicate number of participants with missing data for each variable of interest	4–5
		(c) Summarize follow-up time (e.g., average and total amount)	4–5
Outcome data	15 *	Report numbers of outcome events or summary measures over time	5–7
Main results	16	(a) Give unadjusted estimates and, if applicable, confounder-adjusted estimates and their precision (e.g., 95% confidence interval). Make clear which confounders were adjusted for and why they were included	6–9
		(b) Report category boundaries when continuous variables were categorized	8–9
		(c) If relevant, consider translating estimates of relative risk into absolute risk for a meaningful time period	8–9
Other analyses	17	Report other analyses done—e.g. analyses of subgroups and interactions, and sensitivity analyses	9
		Discussion	
Key results	18	Summarize key results with reference to study objectives	10
Limitations	19	Discuss limitations of the study, taking into account sources of potential bias or imprecision. Discuss both direction and magnitude of any potential bias	11–12
Interpretation	20	Give a cautious overall interpretation of results considering objectives, limitations, multiplicity of analyses, results from similar studies, and other relevant evidence	11–12
Generalizability	21	Discuss the generalizability (external validity) of the study results	12
		Other information	
Funding	22	Give the source of funding and the role of the funders for the present study and, if applicable, for the original study on which the present article is based	13

* Give information separately for exposed and unexposed groups.
